# Genetic diversity among cultivated beets (*Beta vulgaris*) assessed via population-based whole genome sequences

**DOI:** 10.1186/s12864-020-6451-1

**Published:** 2020-03-02

**Authors:** Paul Galewski, J. Mitchell McGrath

**Affiliations:** 1 Department of Plant, Soil, and Microbial Science, Plant Breeding, Genetics, and Biotechnology Program, Michigan State University, 1066 Bogue Street, East Lansing, MI 48824 USA; 2 USDA-ARS, Sugarbeet and Bean Research Unit, 1066 Bogue Street, 494 PSSB, East Lansing, MI 48824 USA

**Keywords:** Sugar beet, Table beet, Fodder beet, Leaf beet, Chard, Genome wide analysis, Crop diversity, Crop differentiation

## Abstract

**Background:**

Diversification on the basis of utilization is a hallmark of *Beta vulgaris* (beet), as well as other crop species. Often, crop improvement and management activities are segregated by crop type, thus preserving unique genome diversity and organization. Full interfertility is typically retained in crosses between these groups and more traits may be accessible if the genetic basis of crop type lineage were known, along with available genetic markers to effect efficient transfer (e.g., via backcrossing). *Beta vulgaris* L. (2n =18) is a species complex composed of diverged lineages (e.g., crop types), including the familiar table, leaf (chard), fodder, and sugar beet crop types. Using population genetic and statistical methods with whole genome sequence data from pooled samples of 23 beet cultivars and breeding lines, relationships were determined between accessions based on identity-by-state metrics and shared genetic variation among lineages.

**Results:**

Distribution of genetic variation within and between crop types showed extensive shared (e.g. non-unique) genetic variation. Lineage specific variation (e.g. apomorphy) within crop types supported a shared demographic history within each crop type, while principal components analysis revealed strong crop type differentiation. Relative contributions of specific chromosomes to genome wide differentiation were ascertained, with each chromosome revealing a different pattern of differentiation with respect to crop type. Inferred population size history for each crop type helped integrate selection history for each lineage, and highlighted potential genetic bottlenecks in the development of cultivated beet lineages.

**Conclusions:**

A complex evolutionary history of cultigroups in *Beta vulgaris* was demonstrated, involving lineage divergence as a result of selection and reproductive isolation. Clear delineation of crop types was obfuscated by historical gene flow and common ancestry (e.g. admixture and introgression, and sorting of ancestral polymorphism) which served to share genome variation between crop types and, likely, important phenotypic characters. Table beet was well differentiated as a crop type, and shared more genetic variation within than among crop types. The sugar beet group was not quite as well differentiated as the table beet group. Fodder and chard groups were intermediate between table and sugar groups, perhaps the result of less intensive selection for end use.

## Background

*Beta vulgaris* L. (beet) is an economically important plant species consisting of several distinct cultivated lineages (*B. vulgaris subsp. vulgaris*) These lineages, or “crop types,” include sugar beet, table beet, fodder beet, and chard. The crop types have been adapted for specific end uses and thus exhibit pronounced phenotypic differences. Crop type lineages breed true, indicating a genetic basis for these phenotypes. Cultivated beets likely originated from wild progenitors of *B. vulgaris subsp. maritima*, also called “sea beet” [5]. It is widely accepted that beet populations were first consumed for leaves. The earliest evidence for lineages with expanded roots occurs in Egypt around 3500 BC. The root types and the origin of the enlarged root is thought to have occurred in the Near East (Iraq, Iran, and Turkey) and spread west (Europe) [50]. Interestingly, beet production for roots as an end use was first described along trade routes across Europe. Historically, Venice represented a major European market of the Silk Road and facilitated the distribution of eastern goods across Europe [24]. Table beet has been proposed to have been developed within Persian and Assyrian gardens [21]. Whether this specifically corresponds to the origin of the expanded root character or a restricted table beet phenotype remains unknown. In fact, early written accounts regarding the use of root vegetables often confused beet with turnip (*Brassica rapa*).

Hybridization between diverged beet lineages has long been recognized as a source of genetic variability available for the selection of new crop types and improving adaptation ([42] cited in [10, 49]). In 1747, Margraff was the first to recognize the potential for sucrose extraction from beet. Achard, a student of Margraff, was the first to describe specific fodder lineages that contained increased quantities of sucrose and the potential for an economically viable source of sucrose for commoditization [49]. In 1787, Abbe de Commerell suggested red mangle (fodder) resulted from a red table beet/chard hybrid and that the progenitors of sugar beet arose from hybridizations between fodder and chard lineages [17, 18]. Louise de Vilmorin (1816–1860), a French plant breeder, first detailed the concept of progeny selection in sugar beet, a method of evaluating the genetic merit of lineages based on progeny performance [20]. Vilmorin used differences in specific gravity as a measure to select beet lineages and increase sucrose content. This approach led to increases in sucrose concentration from ~ 4% in fodder beet to ~ 18% in current US hybrids (reviewed in [35]).

*B. vulgaris* is a diploid organism (2n = 18) with a predicted genome size of 758 Mb [4]. Chromosomes at metaphase exhibit similar morphology [39]. The first complete reference genome for *B. vulgaris* (e.g., RefBeet) provided a new perspective regarding the content of the genome (e.g., annotated gene models, repeated sequences, and pseudomolecules) [15]. This research confirmed whole genome duplications and generated a broader view of genome evolution in the Eudicots, Caryophyllales, and *Beta*. The EL10.1 reference genome [19] represents a contiguous chromosome scale assembly resulting from a combination of PacBio long-read sequencing, BioNano optical mapping and Hi-C linking libraries. Together, EL10.1 and RefBeet provide new opportunities for studying the content and organization of the beet genome. Resequencing of important beet accessions has the potential to characterize the landscape of variation and inform recent demographic history of beet, including the development of crop types and other important lineages.

Population genetic inference leveraging whole genome sequencing (WGS) data have proven powerful tools for understanding evolution from a population perspective [8, 29, 43]. Knowledge of the quantity and distribution of genetic variation within a species is critical for the conservation and preservation of genetic resources in order to harness the evolutionary potential required for the success of future beet cultivation. Recent research has revealed the complexity of relationships within *B. vulgaris* crop types [2]. Studies have shown sugar beet is genetically distinct and exhibits reduced diversity compared to *B. vulgaris subsp. maritima*. Geography and environment are major factors in the distribution of genetic variation within sugar beet accessions in the US [33]. Furthermore, spatial and environmental factors were evident in the complex distribution of genetic variation in wide taxonomic groups of *Beta* [1], which include the wild progenitors of cultivated beet.

Here we present a hierarchical approach to characterize the genetic diversity of cultivated *B. vulgaris* using pooled sequencing of accessions representing the crop type lineages. These accessions contain a wide range of phenotypic variation including leaf and root traits, distinct physiological/biochemical variation in sucrose accumulation, water content, and the accumulation and distribution of pigments (e.g., betaxanthin and betacyanin). These phenotypic traits, along with disease resistance traits, represent the major economic drivers of beet production. Developmental genetic programs involved in cell division, tissue patterning, and organogenesis likely underlie the differences in root and leaf quality traits observed between crop types. Improvement for these traits as well as local adaptation and disease resistance occurs at the level of the population. Pooled sequencing provides a means to characterize the diversity of important beet lineages and survey the nucleotide variation, which has utility in marker-based approaches across a diverse community of breeders and researchers interested in *B. vulgaris*. Pooled sequencing works in synergy with both the reproductive biology of the crop as well as the means by which phenotypic diversity is evaluated (e.g., population mean phenotype) and beets are improved through selection. The genetic control of important beet traits, currently unknown, will help prioritize existing variation and access novel sources of trait variation in order to address the most pressing problems related to crop productivity and sustainability.

## Results

**Fig. 1:**
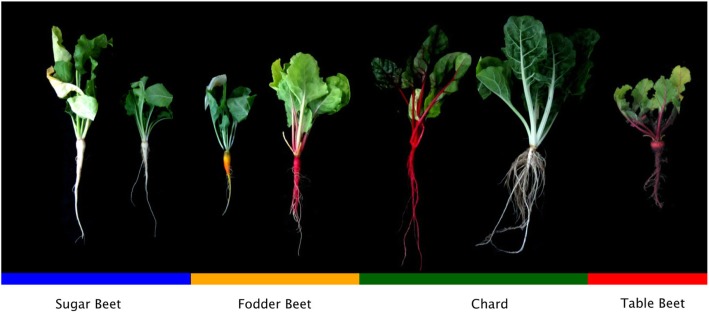
Phenotypes of *B. vulgaris* showing crop type characteristics are distinguishable by 9-weeks of age. Color bars refer to crop type in subsequent figures

Twenty-five individuals from each of the 23 *B. vulgaris* accessions were chosen to represent the cultivated *B. vulgaris* crop types (Table 1 and Fig. 1). Leaf tissue was pooled, DNA extracted and sequenced using the Illumina 2500 in paired end format. On average, 61.84 ± 12.22 GB of sequence data was produced per accession, with an average depth of 81.5X. After processing for quality, reads were aligned to the EL10.1 reference genome. Approximately 20% of bases were discarded owing to trimming of low-quality base calls and adapter sequences. Biallelic SNP and lineage-specific variants were used to estimate the quantity and organization of genome-wide variation within these *B. vulgaris* populations and groups (e.g., species, crop types, and accessions). On average 90.74% of the filtered reads aligned to the EL10.1 reference genome. A total of 14,598,354 variants were detected across all accessions, and 12,411,164 (85.0%) of these were classified as a SNP, and of these 10,215,761 (82.3%) were biallelic. Thus, most SNP variants appeared to be biallelic, as only 2,718,205 (18.6%) of the SNP variants were characterized as multiallelic. After filtering for read depth (*n* ≥ 15), 8,461,457 biallelic SNPs remained for computational analysis. Insertions and deletions (indels) were called using GATK (370,260) (Table 2), which served to reduce false variants resulting from misalignments. This represented a large reduction from the 2,187,190 indels called using the bcftools pipeline.

**Table 1:** List of materials for sequencing

Crop Type	Entry	Accession	Pop ID	PI # / Source	NCBI BioSample	Total Reads	Gb	Coverage (X)	Year Released	Description^a^
Sugar Beet	1	EL10	EL10	689015	SAMN08040263	447,211,041	111.8	149.1	2018	Reference genome assembly short-read set
	2	C869	C869	628754	SAMN12674956	549,262,696	68.7	90.6	2002	Parent population of EL10
	3	EL50/2	EL50	598073	SAMN12842344	487,259,716	60.9	80.4	1994	Cercospora Resistance
	4	EL51	EL51	598074	SAMN12842345	456,623,952	57.1	75.3	2000	Rhizoctonia Resistance
	5	East Lansing Breeding Population	GP10	-	SAMN12842346	492,970,286	61.6	81.3	Pending	OP Recurrent Selection Population
	6	East Lansing Breeding Population	GP9	-	SAMN12842348	847,319,042	105.9	139.7	Pending	OP Recurrent Selection Population
	7	L19	L19	590690	SAMN12842351	767,383,878	76.7	101.2	1978	High Sucrose (>20%)
	8	SP6322	SP7322	615525	SAMN12842349	549,262,696	68.7	90.6	1973	Adaptation to Eastern US
	9	SR102	SR102	675153	SAMN12842347	462,483,116	57.8	76.3	2016	Smooth Root/Low Tare
	10	SR98/2	SR98/2	655951	SAMN12842350	482,270,894	60.3	79.5	2011	Rhizoctonia Resistance
Table Beet	11	Bulls Blood Table Beet	BBTB	Chriseeds	SAMN12842352	519,832,300	65.0	85.7	1700	Historic ornamental and vegetable variety
	12	Crosby Egyptian Table Beet	Crosby	Chriseeds	SAMN12842353	466,455,846	58.3	76.9	1869	US variety with Egyptian background
	13	Detroit Dark Red Table Beet	DDTB	Chriseeds	SAMN12842357	473,659,992	59.2	78.1	1892	US variety
	14	Ruby Queen Table Beet	RQ	Chriseeds	SAMN12842354	500,356,022	62.5	82.5	1950	Current production
	15	Touch Stone Gold Table Beet	TG	Chriseeds	SAMN12842355	396,335,036	49.5	65.4	Unknown	Golden Root
	17	Wisconsin Breeding Line	W357B	Univ. WI	SAMN12842358	538,981,844	53.9	71.1	1982	Self-fertile O-type
	16	Albino Table Beet	WT	Chriseeds	SAMN12842356	503,139,454	62.9	83.0	Unknown	White root
Fodder Beet	18	Mammoth Red Fodder	MAM	Burpee	SAMN12842363	400,297,680	40.0	52.8	1800	Heirloom fodder beet variety
	19	Wintergold Fodder	WGF	Local stock	SAMN12842364	545,378,784	54.5	71.9	Unknown	Winter beet with gold skin pigment
Chard	20	Fordhook Giant	FGSC	Chriseeds	SAMN12842359	484,646,866	60.6	79.9	1934	Green chard
	21	Lucullus Chard	LUC	Chriseeds	SAMN12842361	617,051,314	61.7	81.4	Pre-1700s	Historic green chard variety
	22	Rhubarb Swiss Chard	RHU	Chriseeds	SAMN12842362	538,577,146	53.9	71.1	1857	Red chard
	23	Vulcan Swiss Chard	Vulcan	Chriseeds	SAMN12842360	547,992,902	68.5	90.4	Unknown	Red chard

^a^*OP* open pollinated

**Table 2:** SNP and Indel variation in cultivated *B. vulgaris.* Gene diversity (*2pq*) indicates the diversity and expected genetic variation within populations

Grouping	Accession	Entry	Variation Detected			Lineage-specific Variation			Gene diversity
			Total variants	SNP variants	Indel variants	Total variants	SNP variants	Indel variants	2pq
Sugar Beet	EL10	1	221,493	204,260	17,233	1,149	689	460	0.027
	C869	2	3,479,100	3,147,716	331,384	9,514	8,290	1,224	0.194
	EL50	3	4,226,613	3,805,108	421,505	30,712	27,667	3,045	0.159
	EL51	4	4,222,688	3,808,158	414,530	17,464	15,547	1,917	0.195
	GP10	5	4,070,438	3,689,994	380,444	9,051	7,999	1,052	0.230
	GP9	6	4,216,268	3,803,842	412,426	6,094	5,366	728	0.253
	L19	7	3,492,804	3,185,964	306,840	19,938	17,854	2,084	0.187
	SP7322	8	4,295,147	3,881,458	413,689	15,528	13,942	1,586	0.213
	SR102	9	4,052,933	3,675,246	377,687	8,765	7,846	919	0.232
	SR98	10	4,097,388	3,702,432	394,956	16,241	14,612	1,629	0.202
Table Beet	BBTB	11	4,548,634	4,064,552	484,082	88,129	79,236	8,893	0.087
	Crosby	12	4,553,826	4,112,797	441,029	21,882	19,436	2,446	0.198
	DDRT	13	4,526,694	4,081,640	445,054	24,180	21,592	2,588	0.185
	RQ	14	4,465,888	4,011,300	454,588	31,786	28,714	3,072	0.154
	TG	15	4,066,177	3,655,695	410,482	37,213	33,887	3,326	0.103
	W357B	16	4,096,676	3,674,030	422,646	81,786	74,941	6,845	0.043
	WT	17	4,440,187	3,995,032	445,155	30,371	27,613	2,758	0.159
Fodder Beet	MAM	18	3,366,421	3,087,403	279,018	11,969	10,716	1,253	0.221
	WGF	19	4,286,092	3,887,565	398,527	25,210	22,850	2,360	0.202
Chard	FGSC	20	5,355,215	4,845,307	509,908	31,764	28,455	3,309	0.241
	LUC	21	5,228,873	4,745,987	482,886	35,097	31,341	3,756	0.240
	RHU	22	4,500,515	4,079,774	420,741	29,089	26,138	2,951	0.195
	Vulcan	23	4,852,749	4,378,335	474,414	37,056	33,650	3,406	0.190
Crop Type	Sugar (Entries 1-10)		9,015,627	8,022,713	992,914	3,659	3,317	342	0.207 ± 0.002
	Table (Entries 11-17)		8,871,075	7,875,142	995,933	1,937	1,379	558	0.147 ± 0.044
	Fodder (Entries 18-19)		5,422,289	4,920,209	502,080	848	643	205	0.221 ± 0.013
	Chard (Entries 20-23)		8,684,866	7,788,799	896,067	4,217	3,359	858	0.216 ± 0.027
*B. vulgaris (cultivated)*	*B. vulgaris (SamTools)*		14,598,354	12,411,164	2,187,190	n/a	n/a	n/a	0.182 ± 0.040
	*B. vulgaris (GATK)*		4,180,197	3,809,937	370,260	n/a	n/a	n/a	0.178 ± 0.060

AMOVA was performed in order to quantify the distribution of variation within and among cultivated *B. vulgaris* crop types. The results showed no strong population subdivision with respect to crop type. The variation shared among crop types (99.37%), far exceeded the variation apportioned between crop type lineages (0.40%). The variation detected between accessions within a crop type was also low (0.23%) (Table 3). This result suggested a small proportion of the total variation is unique to any given accession. This was confirmed by the low quantity of lineage-specific variation (LSV) detected, evaluated in a hierarchical fashion. Lineages were defined as individual accessions, crop types, and species (Table 2). In total, 600,239 variants (4.0%) were unique and fixed within a single accession. The accumulation of variation on specific chromosomes for each accession was informative (Table 4). Individual accessions of sugar beet contained a large quantity of LSV on Chromosome 6 relative to other sugar beet chromosomes and indicated that either divergent selection or drift has occurred on this sugar beet chromosome. The variety, ‘Bulls Blood’ (BBTB), contained the greatest amount of LSV detected, 8893 indels and 79,236 SNP variants (Table 2). Table beet accessions contained the most LSV overall which suggested Table Beet is the most divergent of the crop type (Table 4).

**Table 3:** Analysis of molecular variance (AMOVA)

Variance components	Sigma	%
Between Crop Type	0.005	0.40
Within Crop Type	0.003	0.23
Between accessions	1.266	99.37
Total variation	1.274	100

**Table 4:** Number of lineage-specific SNP and indel variants along chromosomes

Crop Type	Pop ID	Entry	Chr 1	Chr 2	Chr 3	Chr 4	Chr 5	Chr 6	Chr 7	Chr 8	Chr 9	mean
Sugar Beet	EL10	1	91	170	103	114	96	229	147	95	104	138
	C86925	2	680	562	1,547	933	2,365	1,101	482	1,316	528	1,057
	EL50	3	1,482	1,496	5,328	2,414	5,141	4,722	3,356	4,244	2,529	3,412
	EL51	4	978	2,436	1,852	1,830	2,019	3,361	1,825	1,772	1,391	1,940
	GP10	5	398	787	964	642	776	2,376	1,331	1,116	661	1,006
	GP9	6	491	521	864	1,023	892	1,839	821	1,028	510	888
	L19	7	568	1,248	993	4,438	845	5,175	3,374	1,918	1,379	2,215
	SP7322	8	467	1,190	1,696	2,026	1,475	4,125	1,906	1,601	1,042	1,725
	SR102	9	406	683	1,081	1,115	1,000	1,458	1,021	1,368	633	974
	SR98	10	419	1,356	1,364	2,056	3,158	3,757	1,423	1,691	1,017	1,805
Table Beet	BBTB	11	17,632	10,425	8,148	9,559	12,067	9,383	4,597	6,131	10,187	9,792
	Crosby	12	2,210	1,172	2,772	2,584	2,511	3,857	2,470	2,548	1,758	2,431
	DDRT	13	2,175	1,314	2,874	3,007	1,776	4,559	4,431	2,195	1,849	2,687
	RQ	14	3,186	3,402	3,680	2,937	4,053	5,349	3,356	3,691	2,132	3,532
	TG	15	3,014	8,486	3,732	3,625	2,971	4,290	3,988	3,716	3,391	4,135
	W357B	16	7,806	4,186	7,661	6,766	16,835	2,011	8,723	5,947	2,102	6,893
	WT	17	3,347	1,577	3,508	4,084	2,777	4,790	3,203	4,876	2,209	3,375
Fodder Beet	MAM	18	698	1,014	885	1,628	1,758	2,820	1,044	1,030	1,092	1,330
	WGF	19	1,014	2,074	4,929	2,468	4,923	4,288	2,041	1,886	1,587	2,801
Chard	FGSC	20	2,883	3,738	2,480	4,665	3,768	4,286	4,181	3,224	2,539	3,529
	LUC	21	2,615	3,570	3,269	3,376	4,834	7,489	4,063	3,118	2,763	3,900
	RHU	22	2,631	2,996	2,249	3,421	2,649	5,019	2,872	3,880	3,372	3,232
	Vulcan	23	3,662	3,977	3,694	4,243	3,343	5,800	3,841	5,054	3,442	4,117
	mean		2,558	2,538	2,855	2,998	3,566	4,003	2,804	2,758	2,096	
Crop Types	Sugar (Entries 1-10)		193	178	2,511	57	71	90	28	990	99	469
	Table (Entries 11-17)		307	53	469	79	292	121	34	528	54	215
	Fodder (Entries 18-19)		69	64	74	49	206	164	41	52	129	94
	Chard (Entries 20-23)		204	826	383	242	610	335	104	660	295	407
	mean		193	280	859	107	295	178	52	558	144	

**Fig. 2:**
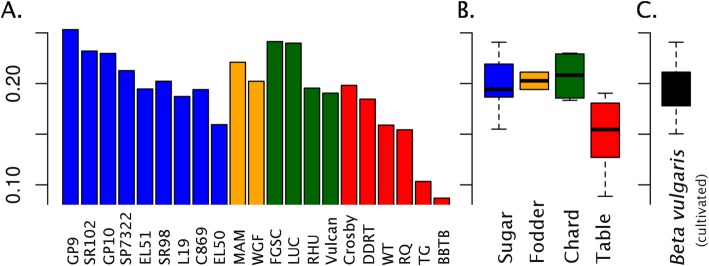
Gene diversity/expected heterozygosity (*2pq*) of *B. vulgaris* lineages. **a** Populations, **b** Crop types, and **c** Species. Colors are coded according to crop type and values are present in Table 2

Within the crop types, 10,661 variants were crop type specific and were not found within any other crop type. Of these, 8098 were characterized as SNPs and 1963 as indels. The number of SNP LSV detected within sugar beet, table beet, fodder beet, and chard crop types were as follows: 3317, 1379, 643, and 3359, respectively (Table 2). Indel LSV detected for the crop types were 342, 558, 205, and 858, respectively (Table 2). Diversity contained within the species, crop type, and individual accessions was estimated using expected heterozygosity (*2pq*) (Table 2 and Fig. 2). Expected heterozygosity (*2pq*) varied from 0.027 in our inbred reference EL10 sugar beet accession to 0.253 in the recurrent selection sugar beet breeding population GP9. Within the crop types, the mean expected heterozygosity for sugar beet was 0.207, table beet = 0.147, fodder beet = 0.221, and chard = 0.216 (Table 2). Interestingly, chard contained the most LSV of the crop types yet showed high diversity (*2pq*), suggesting unique variation supports the divergence of this lineage.

The expected heterozygosity (*2pq*) for accessions such as EL10 and W357B was low. This was expected owing to inbreeding via the presence of self-fertility alleles in these two accessions. These accession EL10 was excluded from further analysis due to the fact that the sequence data was derived from a single individual. Interestingly, the variety ‘Bulls Blood’ lacked variation relative to other beet accessions, and it is likely that recent selection underlies this result (Chris Becker, personal communication). The variation in diversity estimates as measured by expected heterozygosity (*2pq*) suggested the level of diversity is highly dependent on the breeding system, selection for end use traits and Ne size.

**Fig. 3:**
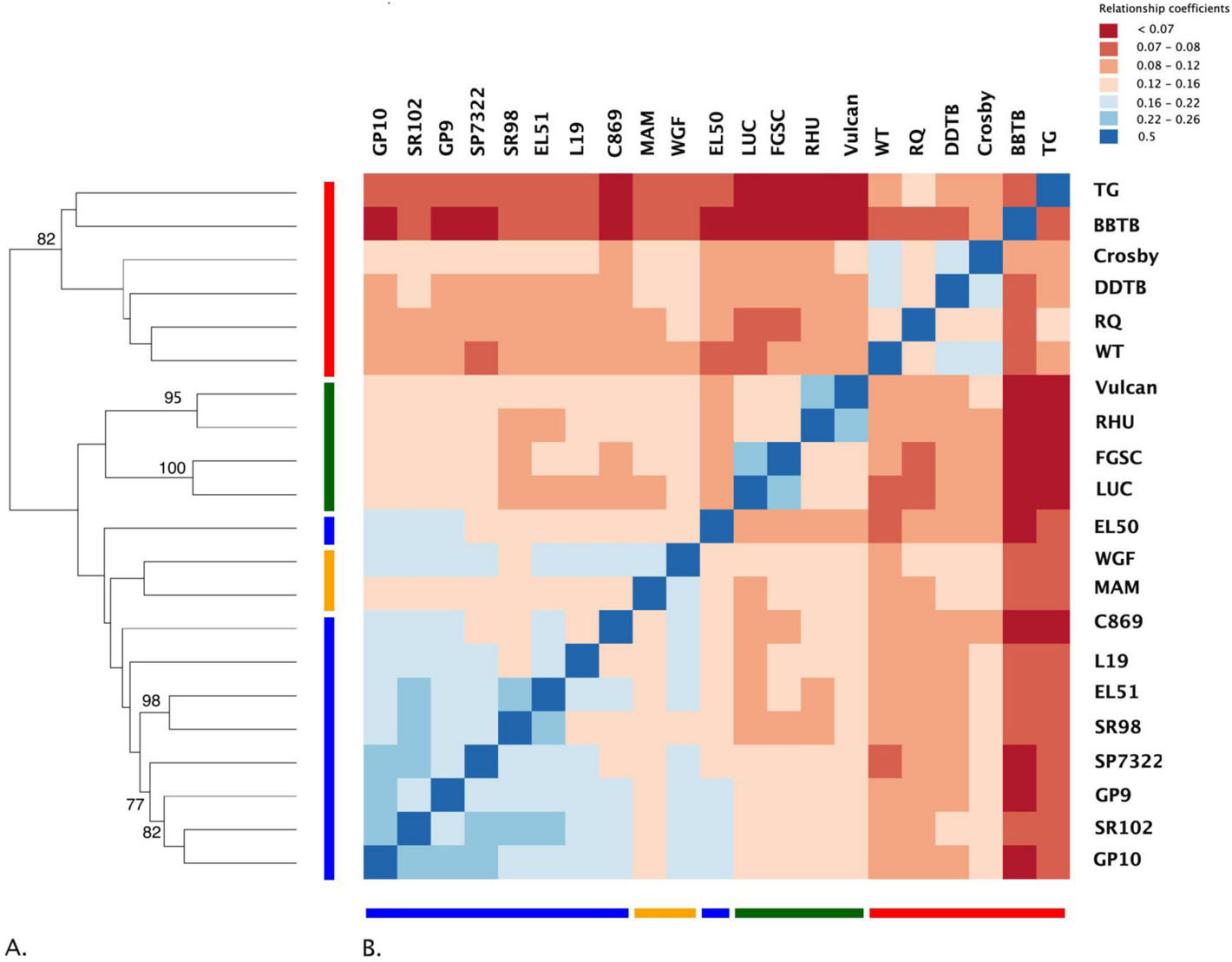
Lineage relationships inferred by hierarchical clustering of pairwise relationship coefficients. **a** Dendrogram reflects support for clusters. Branch lengths indicate relationship coefficients between lineages, high (blue) and low (red). **b** Heatmap shows relationship coefficient values for all comparisons. Colors at the bottom and left of heat map represent crop type, sugar beet (blue), fodder beet (orange), chard (green), table beet (red)

The variation detected was used to cluster accessions in two ways: (1) a hierarchical clustering based on relationship coefficients estimated using the quantity of shared variation between accessions, and (2) a principal components analysis using allele frequency in each accession, estimated using an IBS (Identity by State) approach. The resulting dendrogram and heatmap showed that the table beet crop type was the only group to have strong evidence (e.g., high relationship coefficients and bootstrap values) supporting it as a unique group harboring significant variation (Table 5). Likewise, the green (LUC and FGSC) and red (RHU and Vulcan) chard accessions showed evidence for two distinct groups (Fig. 3). Sugar beet lineages with known pedigree relationships and high probability for shared variation (e.g., SR98/2 and EL51) also had strong evidence, which supports the delineation of population structure on the basis of shared variation. Additionally, the clade composed of SP7322, SR102, GP10, and GP9 resolved in a similar fashion.

**Table 5:**
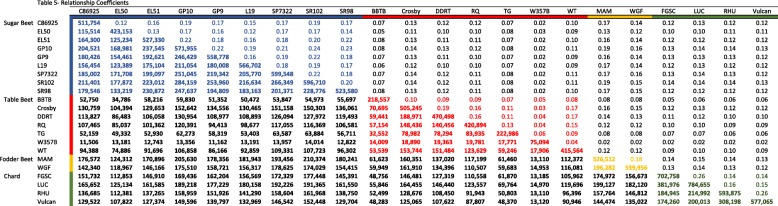
Pairwise relationship matrix. Relationship coefficients are indicated above the diagonal, the number of shared variants is indicated below the diagonal, and the number of variants is given on the diagonal

**Fig. 4:**
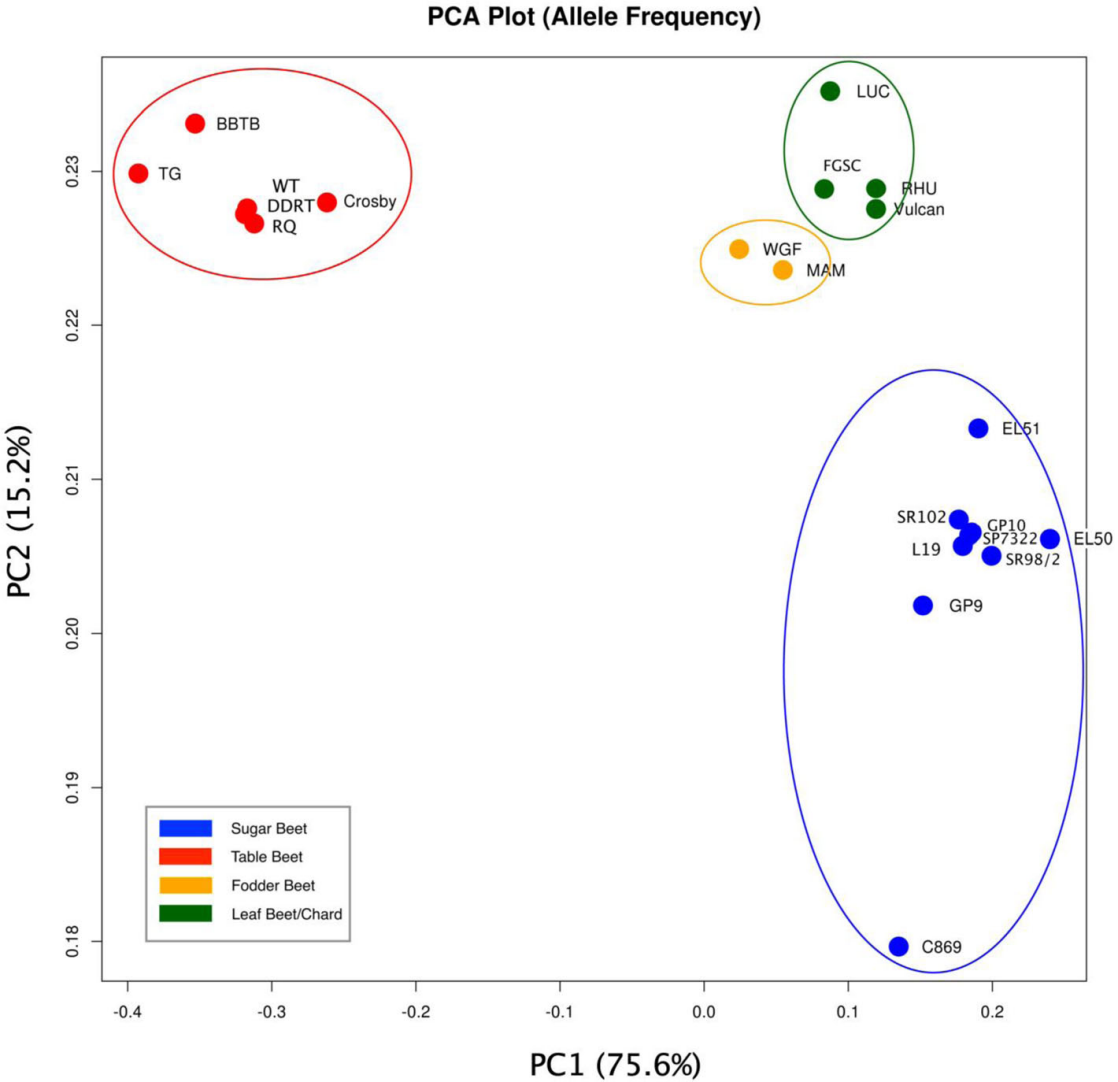
PCA plot showing the separation of crop types using genome-wide allele frequency data

**Fig. 5:**
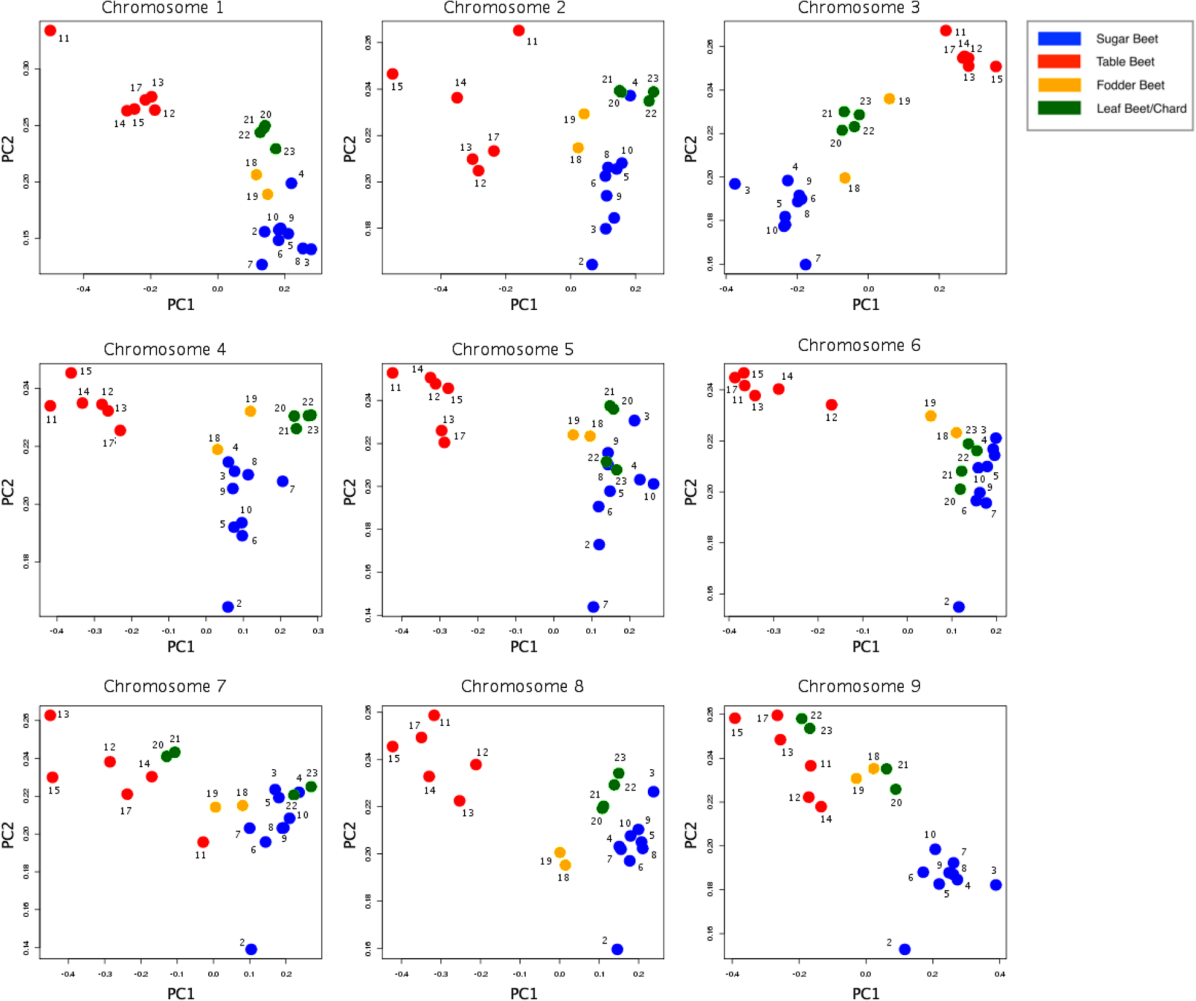
PCA plot showing the separation of crop types using allele frequency data on a chromosome by chromosome basis. Colors group crop types as in Fig. 4

PCA used genome-wide allele frequency estimates for individual accessions. The first principal component (PC1) explained 75.6% of the variance in allele frequency and separated the table beet crop type from the other crop types. The second component (PC2) explained 15.25% of the variance (Fig. 4). Sugar and table beets appeared the most divergent and were able to be separated along both dimensions. Chard and fodder crop types were distinguishable but appeared less divergent. Allele frequency estimates analyzed on a chromosome-by-chromosome basis demonstrated that specific chromosomes cluster the accessions by crop type (Fig. 5). Chromosomes 3, 8, and 9 appear to be important for the divergence between sugar beet and other crop types. All chromosomes were able to separate table beet with the exception of Chromosomes 7 and 9.

**Fig. 6:**
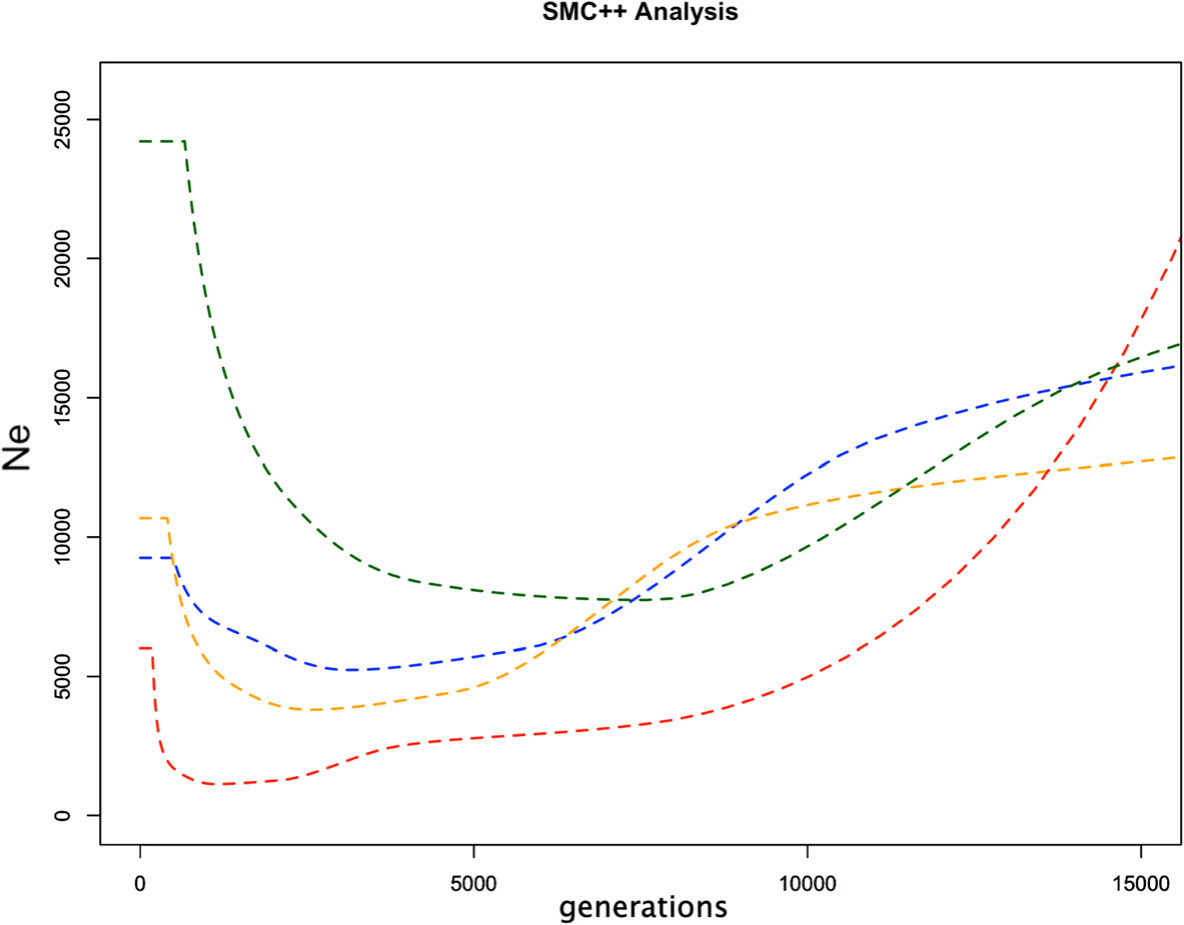
Inferred historical Ne of *B. vulgaris* crop types using the program SMC++. Colors group crop types. Red = table beet, blue = sugar beet, green = chard (leaf beet), yellow = fodder beet

Finally, using our population genomic data we tested a composite likelihood method to estimate historical effective population size (Ne) to infer demographic histories for crop type lineages. Table beet appears to have a distinct history in this respect as well as one or more demographic separations when compared with the other three lineages. Trends in historical effective population sizes (Ne) for fodder and sugar groups were quite similar to each other, and no early divergence was detected between them. The chard group appeared to share early demographic history with the fodder/sugar group but showed a different trend later, suggesting it diverged early with respect to the other crop types (Fig. 6). The demographic history of *B. vulgaris* crop type correlates well with historical evidence (e.g., records of antiquity, archeological evidence, and scientific literature) detailing the development of distinct crop type lineages (Table 6).

**Table 6:** Historical time line highlighting evidence of beet utilization

Date	Source	Description
before 8500 BCE	^a,b^	*B. vulgaris spp maritima gathered as potherbB. vulgaris spp maritima gathered as potherb*
8500 BCE	^a,b^	The domestication of leaf beet in eastern Turkey
3500 BCE	^a^	Leaf and root types present in Egypt
1200 BCE	^a^	Leaf beet present in Syria
1000 BCE	^a^	Leaf beet present in Greece
600 BCE	^a^	Leaf beet present in China
460 BCE	^a^	Black beet mentioned (perhaps a reference to table beet)
250 BCE	^a,b^	Table beet cultivation spreads
50 BCE	^a,b^	*Beta cultivation spreads in Roman EmpireBeta cultivation spreads in Roman Empire*
1,000 – 1300 CE	^a,b^	Beet described as a garden vegetable, with many types.
1500 CE	^a,b^	Fodder beet spreads across Europe
1747 CE	^a,b^	Margraff demonstrates sucrose can be extracted from beet
1800 CE	^a,b^	Achard identifies fodder lineages with potential use as a sugar crop
1816–1850 CE	^a,b^	Vilmorin develops progeny selection to increase sugar content using differences in specific gravity

Dates compiled from the following sources ^a^Biancardi et al. 2012, ^b^Cook and Scott 1993

## Discussion

The accessions sampled here represent divergent lineages used in the cultivation of beet. All have notable breeding histories, which has served to capture and fix genetic variation resulting in predictable phenotypes characteristic of each lineage (e.g. accession or crop type). The organization and distribution of genetic variation within and between accessions reflects the historical selection and evolutionary pressures experienced as these crop types and varieties were developed. Pooled sequencing allowed us to make the cogent genomic comparisons that informs the history of beet development, from ancestral gene pools and domestication to the development of varieties and germplasm within modern breeding programs. Using population genomic data, we were able to support *B. vulgaris* as a species complex, uncover genomic variation associated with development of beet crop types, and gain fundamental insight into the natural history of beet.

Two biological groups could be identified with high confidence using these data, a table beet group and a group encompassing chard, fodder beet, and sugar beet. Previous research, which used genetic markers to cluster crop types, reported similar findings [1, 30]. The strong evidence for a unique table beet group hints at both genetic drift, resulting from reproductive isolation, as well as positive selection for end use (Figs. 3, 4, 6). In general, selection and drift act to change allele frequency within a population [23], but the effects are relative to the effective population size (Ne) of the populations under selection. Effective population size is an important consideration because it relates to the standing genetic diversity within populations (Crow and Denniston [11, 47]). The patterns of variation resulting from drift and selection are distinct. For example, table beet accessions had low diversity (*2pq*) relative to other crop types (Table 2), and the ability to separate table beet accessions on the basis of allele frequency is suggestive of selection (Figs. 4 and 5). Relationship coefficients, on the other hand, highlight the differences in the quantity of shared variation within and between crop types (Table 5 and Fig. 3), suggesting table beet may have been less connected to other crop types historically. Allele frequency showed signals of differentiation distributed across all chromosomes for table beet (Fig. 5), likely reflecting both selection and drift. The low quantity of shared variation between crop types did not support long term phylogeographic explanations for the differentiation observed. Long periods of geographic isolation can produce barriers to reproduction, further reinforcing isolation and divergence of populations [40]. This appears not to be the case in cultivated beet, as experimental hybrids between crop types show few barriers to hybridization and produce viable progeny, which does not suggest a large degree of chromosomal variation between the groups. The creation of segregating populations from crosses between sugar and table beet crop types support this observation [26, 34].

The lesser degree of separation between chard, fodder, and sugar crop types may be the result of increased connectivity (e.g., historical gene flow) between these lineages versus table beet. High gene flow exerts a homogenizing effect on the diversity contained within populations and increases the quantity of shared variation. This may explain a lack of clear delineation of these crop types using genome-wide markers. Fodder and sugar crop types could be separated using allele frequency (Fig. 4) but clusters based on shared variation were less clear (Fig. 3). This was not unexpected given the known history between these lineages. The development of fodder lineages that accumulate sucrose have occurred in recent history (~ 200 years), giving rise to the progenitor of sugar beet, the ‘White Silesian’ [17, 49]. Phenotypic divergence between species is attributed more to indel variation than to SNP variation owing to their greater consequences on gene expression and gene regulation [9]. This phenomenon may be visible in population divergence as well as speciation. The high quantity of shared variation between sugar and fodder crop types (Table 5) and the low quantity of indel LSV detected within sugar and fodder crop types (Table 2) likely reflects a shared demographic history relative to comparisons between other crop types (Fig. 6). Interestingly, chard contained the most LSV of the crop types yet showed high diversity (*2pq*), suggesting some unique variation supports the divergence of this lineage. The larger quantity of shared variation between the sugar beet, fodder beet, and chard crop types versus table beet (Table 5) suggests differences in the extent and timing of gene flow between lineages.

Chard is hypothesized as the first crop type developed from diverse ancestral *B. vulgaris subsp. maritima* populations [5, 49]. This is supported by the high level of diversity (*2pq*) (Table 2 and Fig. 2), a high quantity of LSV (Table 2), and interesting trends in the demographic history (Fig. 6). The clear delineation of two distinct chard groups (Fig. 3) suggests major differences in genome composition between the two groups and a unique demographic history for each chard lineage. The chards share similar leaf morphology but the roots of the red chard group were enlarged and had fewer ‘sprangles’ (e.g. adventitious roots branching from the tap root) relative to the green chard accessions but not to the extent as the root types (e.g. sugar, fodder, and table). This may reflect introgressions between the red chard and a root type, and potentially an unintended consequence of chard improvement for color traits.

The enlarged tap root character appears to have been first developed in table beet lineages [5], but the expanded root character is shared across crop type lineages. This suggests several hypotheses: (1) the root character in fodder beet reflects the introgression of this character from a table beet to a chard background and represents a single source for this character [50], (2) an ancestral population gave rise to the root character and diverged into fodder and table lineages, (3) the enlargement evolved several times and contributes to the diversity in shape and form. Historically, it appears admixture, hybridization, and introgression were fundamental to the development of beet lineages. Schukowsky [42] suggested that the broad adaptation of beet to novel growing environments may be due to variation accumulated in geographically diverse ancestral populations and shared via admixture and gene flow between lineages. Trait variation in wild relatives is becoming increasingly important for crop adaptation to a changing growing environment [44]. Distinguishing between sorting ancestral variation and introgression events remains a challenge in population genomic analysis but could yield important insight into beet crop type development, and other cultivated species as well.

The beet crop types have appeared to have diverged by selection. The variation in allele frequency of bi-allelic SNPs for beet accessions was able to distinguish the crop types (Fig. 4). This suggests that the allele frequency data contains signal related to historical selection (Fig. 5). Sugar and table beet appear to be the most diverged, which is consistent with large breeding efforts for each of these crop types. Allele frequency data analyzed on a per chromosome basis demonstrated that only specific chromosomes can differentiate on the basis crop type. Ostensibly the presence of variation located on specific chromosomes is under positive selection for end use, leading to an accumulation of lineage-specific differences including those linked to defining phenotypic characters. In fact, many quantitative trait loci studies support the fact that specific regions along chromosomes contain the variation that ultimately influences phenotype [14]. Interestingly, even small amounts of variation can have profound effects on phenotypic variation [13, 37]. Allele frequency estimates for specific chromosomes as well as the variation in lineage-specific variation for crop type on specific chromosomes suggests a small degree of total genome variation explains beet crop type differences. Given the support for crop type relationships based on allele frequency and degree of shared variation, it appears the divergence of beet crop types occurred in the presence of high gene flow. Population divergence in the presence of gene flow produces distinct patterns of variation with respect to selection [32]. Cryptic relationships within other species complexes have been explained by various models including the islands-of-differentiation model [6, 48].

Admixture and introgression events may have served to share genetic variation across cultivated beet accessions and crop type lineages, which in turn, created challenges for the clear delineation of subpopulations. This is confounded by the fact that, as lineages evolve, a lesser quantity of variation with greater agricultural importance contributes to our notion of economic and agronomic value. Resolving the degree to which historical admixture and introgression has contributed to the development of beet crop type will require more in-depth analysis of the variation at nucleotide level within local chromosome regions.

## Conclusions

Beet crop types are important lineages which exhibit both genetic and phenotypic divergence. Sufficient support for treatment of these groups as significant biological units was present from de novo clustering of beet accessions. It would appear selection for end use qualities and genetic drift were major factor in the observed differentiation between lineages and explains the apportionment of genetic variation between crop types at distinct chromosome locations. Common ancestry and admixture and introgression likely maintained levels of genetic variation between crop types and reflects a complex demographic history between crop types. The majority of genetic variation detected in beet crop types was biallelic SNPs, but lineage specific variation may have had a greater role in crop diversification, with table beet showing the greatest degree of differentiation. Most variation is held within the species (as represented by the crop types here), and only a small amount of the total variation is partitioned within individual crop types. Understanding the history of beet crop type diversification, in terms of the evolution of genomes and traits within and between crop types, will help to identify and recover a genetic basis for crop type phenotypes. Directed molecular breeding approaches may be developed to incorporate novel traits from other crop types and wild populations.

## Methods

### *Beta vulgaris* accessions and sequencing

Twenty-three beet accessions were sequenced to 80X coverage relative to the predicted 758 Mb *B. vulgaris* genome using a pooled sequencing approach. The accessions are representative of the four recognized crop types and capture the range of phenotypic diversity found within cultivated beet (Table 1). Accessions were grown in the greenhouse and leaf material was harvested from 25 individuals per accession. Leaf material, one young expanding leaf of similar size from each individual within an accession, was combined, homogenized, and DNA was extracted using the Macherey-Nagel NucleoSpin Plant II Genomic DNA extraction kit (Bethlehem, PA). Libraries were prepared using the Illumina TruSeq DNA Nano Library Preparation Kit. Libraries were QC’d and quantified using a combination of Qubit dsDNA HS, Caliper LabChipGX HS DNA and Kapa Biosystems Illumina Library Quantification qPCR assays. Each set of 8 libraries were pooled in equimolar amounts. Each of these pools was loaded on four (4) lanes of an Illumina HiSeq 2500 High Output flow cell (v4). Sequencing was done using HiSeq SBS reagents (v4) in a 2x125bp paired end format. Base calling was performed by Illumina Real Time Analysis (RTA) v1.18.64 and output of RTA was demultiplexed and converted to FastQ format with Illumina Bcl2fastq v1.8.4. The resulting reads were assessed for quality using FastQC [3], library bar-code adapters were removed, and reads were trimmed according to a quality threshold using TRIMMOMATIC [7] invoking the following options (ILLUMINACLIP:adapters.fa-:2:30:10 LEADING:3 TRAILING:3 SLIDINGWINDOW:4:15 MINLEN:36). These filtered reads were used for downstream analysis.

### Data processing and variant detection

Variants for each accession were called by aligning the filtered reads to the EL10.1 reference genome assembly [19] using bowtie2 v2.2.3 (options -q --phred33-quals -k 2 -x) [25]. An insert size distribution was estimated for paired end read mappings (Additional file 1: Figure S1). The resulting alignment files were sorted and merged using SAMtools version 0.1.19 [28]. SNP variants were called for each accession using BCFtools [27], filtered for mapping quality (MAPQ > 20) and read depth (*n* > 15), and then combined using VCFtools [12]. The combined data was again filtered to obtain biallelic sites across all accessions. Indels were evaluated using the Genome Analysis Toolkit (GATK) haplotype caller [36] following best practices (https://gatk.broadinstitute.org/hc/en-us/sections/360007226651-Best-Practices-Workflows). Indel size distribution was also calculated (Additional file 2: Figure S2). The ‘m*pileup*’ subroutine in SAMtools was then used to quantify the alignment files and extract allele counts. Allele frequency was estimated within each accession for SNP loci identified as biallelic across all accessions. Population parameters were then estimated using allele frequencies within each accession such that (*p* + *q* = 1), where p was designated as the allele state of the EL10.1 reference genome and q, the alternate, detected in each sequenced accession. Expected heterozygosity (*2pq*), also termed gene diversity [38], was used to compare diversity contained within each accession.

### AMOVA

Analysis of molecular variance (AMOVA) was used to assess the distribution of genetic variation within the species [16]. AMOVA was performed using the *ade4* package in R [46] following the approach for pooled sequence data outlined in Gompert et al. [22].

### Crop type relationships

Biallelic SNPs were used to calculate pairwise relationship coefficients between accessions using an identity by state (IBS) approach within the Kinship Inference for Association Genetic Studies (KING) package [31]. Neighbor joining trees were generated in order to extract bootstrap support along branches of our phylogram. In total 100 replications were used and analysis was carried out using the ape package (Analyses of Phylogenetics and Evolution) in R (Paradis and Schliep [41]).

### Principle components analysis (PCA)

PCA was carried out in R using singular value decomposition function, svd() in R.

### Population size history

Composite likelihood methods were used to estimate historical population sizes and infer demographic history from genome sequences of each accession using the program SMC++ version 1.12.1 [45] invoking the commands (smc++ estimate -o analysis/ 1.25e-8) to estimate historical population size and (smc++ split -o split/ pop1/model.final.json pop2/model.final.json) to estimate the joint demography between populations. A mutation rate of 1.25e-8 was assumed used based on the Arabidopsis mutation rate predicted to be between 10e-7 and 10e-8.

### Lineage-specific variation

Lineage-specific variation (LSV), defined as homozygous private variation (e.g., apomorphy), was extracted from the merged VCF file containing variants for all accessions. Variants that were fixed within a particular accession or assemblage of accessions (lineage), and not detected within any other lineage, were considered LSV. Variant files representing LSV were produced for each lineage in a hierarchical fashion (e.g., species, crop type and accessions). LSV was then evaluated with respect to lineage as well as its distribution along chromosomes.

## Supplementary information

**Additional file 1 : Figure S1.** Insert size distribution for PE sequencing libraries for *B. vulgaris* accession C869. (max = 64,496,131, min = 32, median = 440, and standard deviation = 511,068)

**Additional file 2 : Figure S2.** Size distribution for indels detected within cultivated *B. vulgaris* accessions.

## Data Availability

The datasets used and/or analyzed during the current study are available from the corresponding author on reasonable request. Whole genome sequences for the reported accessions have been deposited in NCBI under the BioProject Accessions PRJNA563463 (population whole genome sequences) and PRJNA413079 (EL10 genome assembly). Code available at https://github.com/BetaGenomeNinja/BMC_Genetic-diversity_Beets and data sets including vcf files and the allele frequency matrix is available via Data Dryad (10.5061/dryad.sbcc2fr2t).

## Abbreviations

*2pq*: Expected heterozygosity
AMOVA: Analysis of molecular variance
GATK: Genome analysis toolkit
IBS: Identity by State
Indel: Insertion/deletion
LSV: Lineage specific variation
Ne: Effective population size
PCA: Principle components analysis
SNP: Single nucleotide polymorphism
WGS: Whole genome sequencing
