# Supplementary material for: Genetic diversity among cultivated beets (Beta vulgaris) assessed via population-based whole genome sequences
Source: BMC Genomics. 2020 Mar 2;21:189. doi: 10.1186/s12864-020-6451-1 (PMC7053042; doi:10.1186/s12864-020-6451-1)
Supplement: Supplementary file 1 [file 12864_2020_6451_MOESM1_ESM.docx]

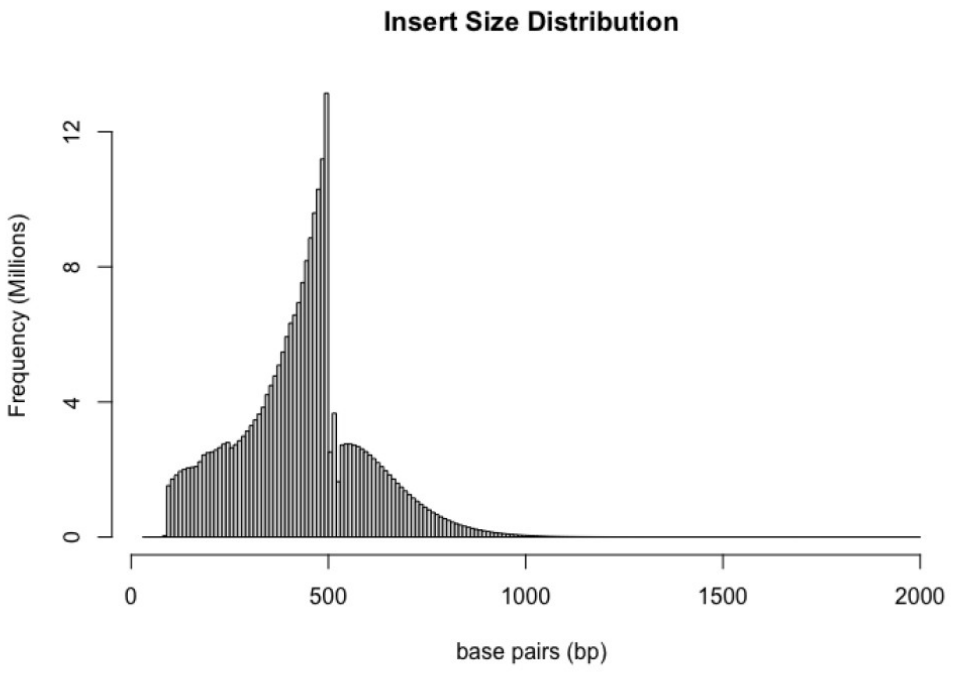
Insert Size Distribution
Frequency (Millions)
0
4
8
12
0
500
1000
1500
2000
base pairs (bp)
