# Supplementary material for: Genetic diversity among cultivated beets (Beta vulgaris) assessed via population-based whole genome sequences
Source: BMC Genomics. 2020 Mar 2;21:189. doi: 10.1186/s12864-020-6451-1 (PMC7053042; doi:10.1186/s12864-020-6451-1)
Supplement: Supplementary file 2 [file 12864_2020_6451_MOESM2_ESM.docx]

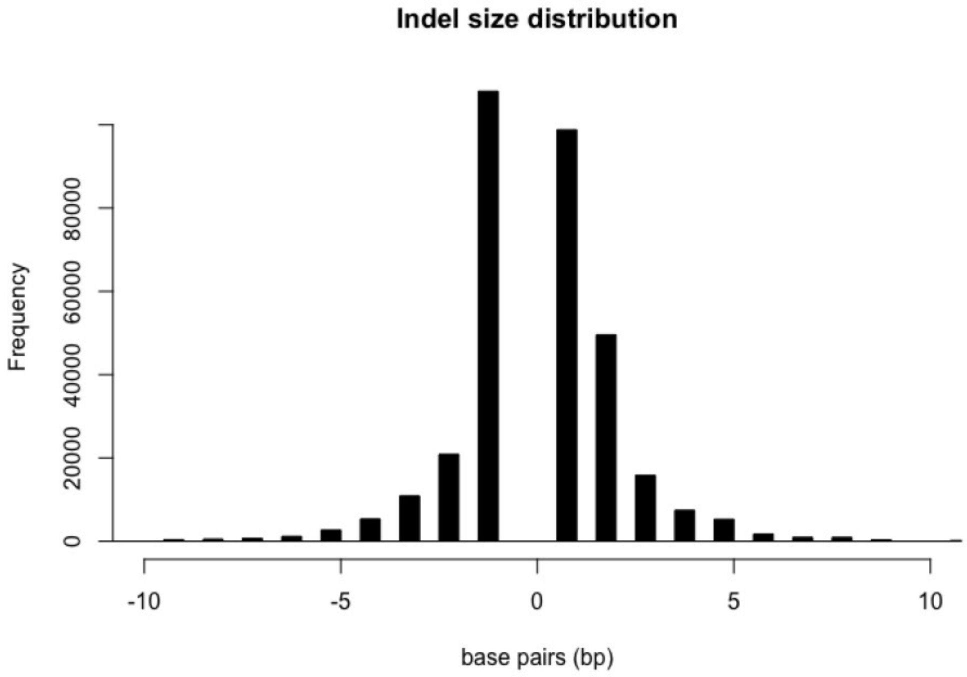

Indel size distribution
Frequency
0
20000
40000
60000
80000
-10
-5
0
5
10
base pairs (bp)
